# Supplementary material for: Association between AHR Expression and Immune Dysregulation in Pancreatic Ductal Adenocarcinoma: Insights from Comprehensive Immune Profiling of Peripheral Blood Mononuclear Cells
Source: Cancers (Basel). 2023 Sep 19;15(18):4639. doi: 10.3390/cancers15184639 (PMC10526859; doi:10.3390/cancers15184639)
Supplement: Supplementary file 1 [file cancers-15-04639-s001.zip › cancers-2612093- Table S1.pdf]

Supplementary Materials

Table S1. Clinical–pathological parameters of the subjects studied.

	Pancreatic Cancer	Healthy Controls
**Number**	30	30
**Gender (Male:Female)**	19:11	7:23
**Mean age (range in years)**	61.7 (37–87)	50.8 (23–78)
**Tumour size pT**		
1	0	
2	8	
3	10	
4	12	
**pN-category**		
pN0	6	
pN1	6	
pN2	10	
pN+	8	
**Distant metastasis**		
No	23	
Yes	7	
**UICC stage**		
I	1	
II	11	
III	11	
IV	7	
**Radical surgery**		
No	15	
Yes	15	
