# Supplementary material for: Association between AHR Expression and Immune Dysregulation in Pancreatic Ductal Adenocarcinoma: Insights from Comprehensive Immune Profiling of Peripheral Blood Mononuclear Cells
Source: Cancers (Basel). 2023 Sep 19;15(18):4639. doi: 10.3390/cancers15184639 (PMC10526859; doi:10.3390/cancers15184639)
Supplement: Supplementary file 1 [file cancers-15-04639-s001.zip › cancers-2612093-File S1.pdf]

1. **PVDF MEMBRANE**

P110, P113, C517,C404, C507 - GAPDH blots were used for Figure 5a and 8a.

P108 was excluded for wrong diagnose.

C403 and C514 were excluded for other diseases.

RAW 264.7 was used for positive control.

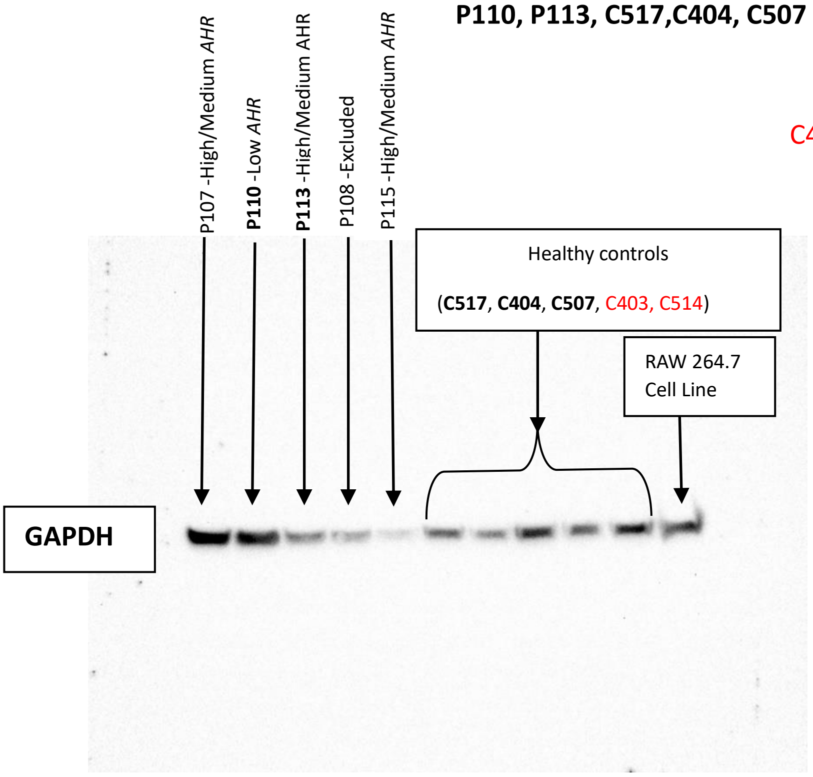

P110, P113, C517,C404, C507 - AHR blots were used for Figure 5a.

P108 was excluded for wrong diagnose.

P108 was excluded for wrong diagnose.

C403 and C514 were excluded for other diseases.

RAW 264.7 was used for positive control

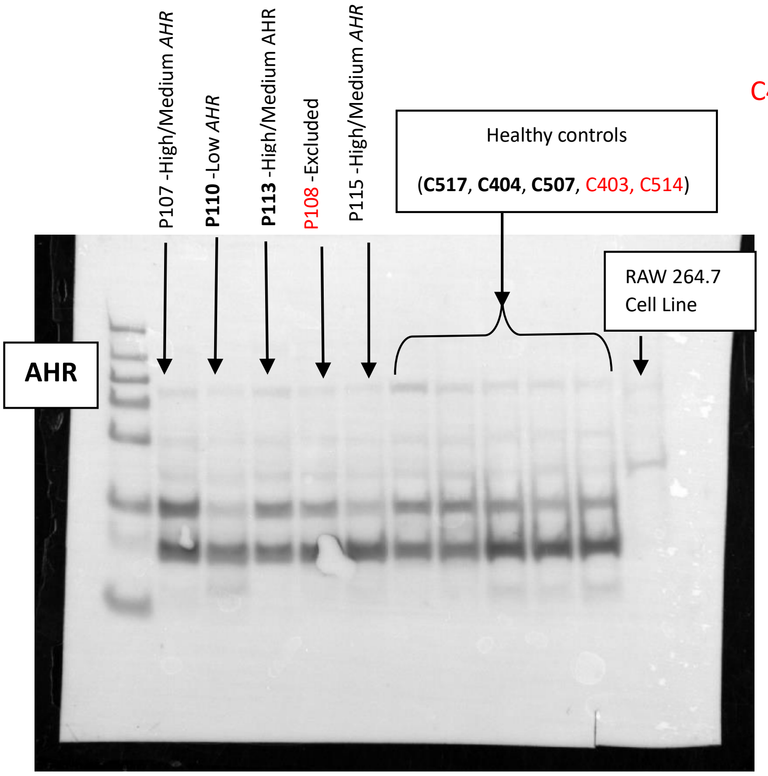

P110, P113, C517,C404, C507

PD1 blots were used for Figure 8a.

P108 was excluded for wrong diagnose.

C403 and C514 were excluded for other diseases.

RAW 264.7 was used for positive control

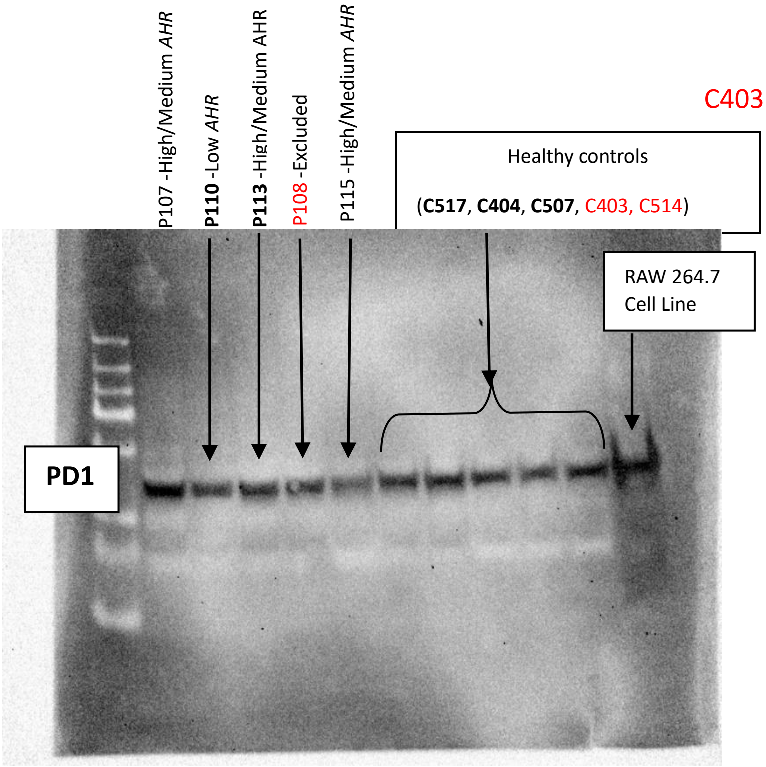

2. PVDF MEMBRANE was used only for normalised analysis.

P124 was excluded for wrong diagnose.

C401, C510, C515 were excluded for other diseases

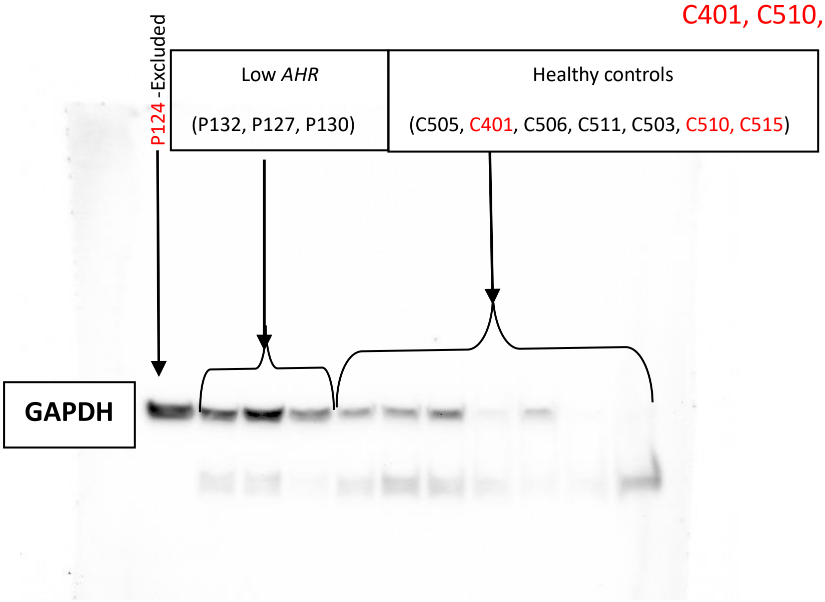

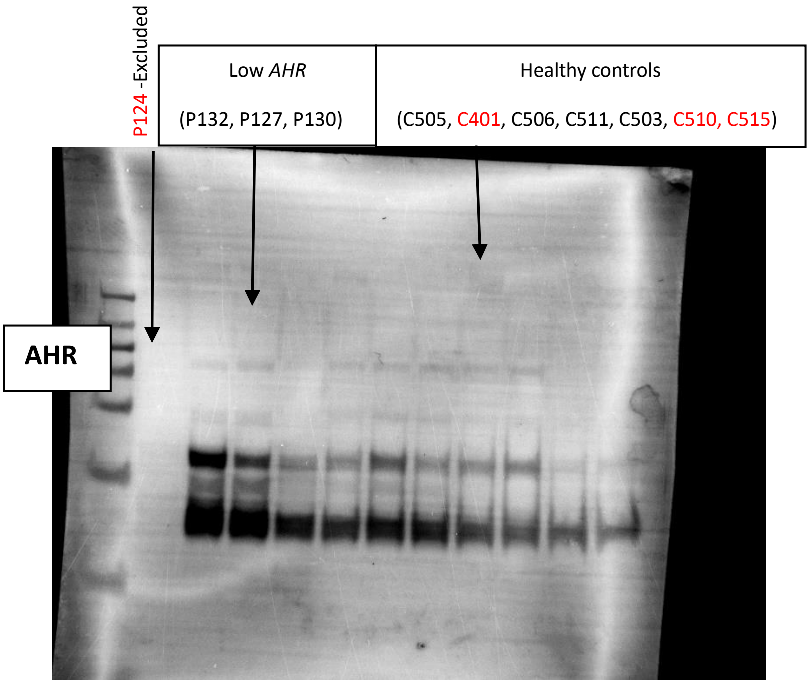
P124 -Excluded
Low *AHR*
(P132, P127, P130)
Healthy controls
(C505, C401, C506, C511, C503, C510, C515)
AHR

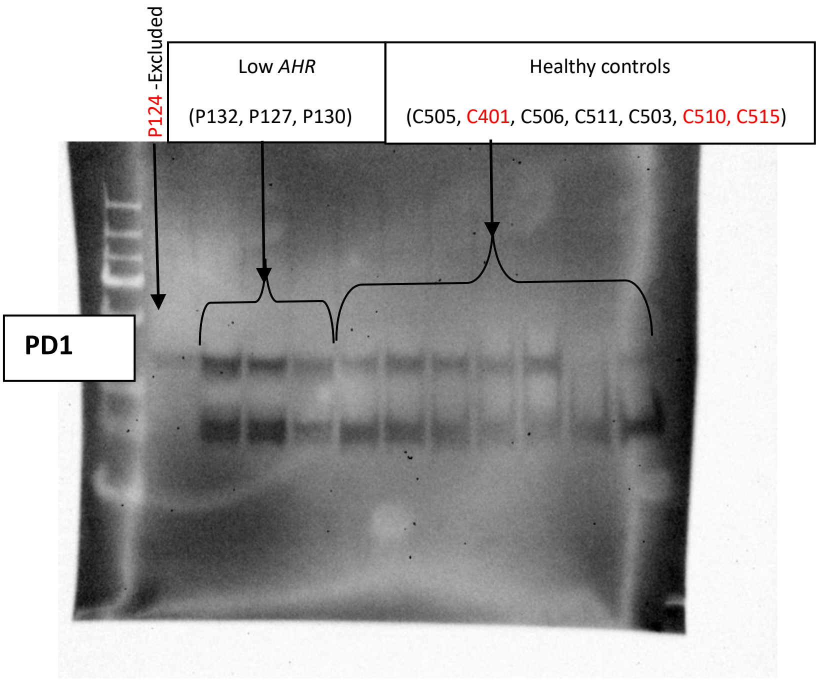
P124 -Excluded
Low *AHR*
(P132, P127, P130)
Healthy controls
(C505, C401, C506, C511, C503, C510, C515)
PD1
